# Household members do not contact each other at random: implications for infectious disease modelling

**DOI:** 10.1098/rspb.2018.2201

**Published:** 2018-12-12

**Authors:** Nele Goeyvaerts, Eva Santermans, Gail Potter, Andrea Torneri, Kim Van Kerckhove, Lander Willem, Marc Aerts, Philippe Beutels, Niel Hens

**Affiliations:** 1 Interuniversity Institute for Biostatistics and Statistical Bioinformatics, UHasselt, Hasselt, Belgium; 2 The Emmes Corporation, Rockville, MD, USA; 3 Centre for Health Economics Research and Modelling Infectious Diseases, Vaccine and Infectious Disease Institute, University of Antwerp, Antwerp, Belgium

**Keywords:** epidemic model, household contact network, ERGM, random mixing, infectious disease

## Abstract

Airborne infectious diseases such as influenza are primarily transmitted from human to human by means of social contacts, and thus easily spread within households. Epidemic models, used to gain insight into infectious disease spread and control, typically rely on the assumption of random mixing within households. Until now, there has been no direct empirical evidence to support this assumption. Here, we present the first social contact survey specifically designed to study contact networks within households. The survey was conducted in Belgium (Flanders and Brussels) from 2010 to 2011. We analysed data from 318 households totalling 1266 individuals with household sizes ranging from two to seven members. Exponential-family random graph models (ERGMs) were fitted to the within-household contact networks to reveal the processes driving contact between household members, both on weekdays and weekends. The ERGMs showed a high degree of clustering and, specifically on weekdays, decreasing connectedness with increasing household size. Furthermore, we found that the odds of a contact between older siblings and between father and child are smaller than for any other pair. The epidemic simulation results suggest that within-household contact density is the main driver of differences in epidemic spread between complete and empirical-based household contact networks. The homogeneous mixing assumption may therefore be an adequate characterization of the within-household contact structure for the purpose of epidemic simulations. However, ignoring the contact density when inferring based on an epidemic model will result in biased estimates of within-household transmission rates. Further research regarding the implementation of within-household contact networks in epidemic models is necessary.

## 1. Introduction

Households are crucial units in the epidemiology of airborne infectious diseases such as influenza, smallpox and SARS. Relations between household members are typically characterized by frequent and intimate contacts, allowing for rapid disease spread within the household upon introduction of an infectious case. As stated by Ferguson *et al.* [1], ‘being a member of a household containing an influenza case is in fact the largest single risk factor for being infected oneself’ (p. 450, citing [2,3]). Furthermore, households with children have a bridging function, allowing for an infection to spread from schools to workplaces, and vice versa. Inference from household final size data revealed that children play a key role in bringing influenza infection into the household and in transmitting the infection to other household members [3]. Households are the most common transmission unit used in observational studies and in epidemic models.

Many epidemic models rely on the assumption of homogeneous (random) mixing within households. In early work, the Reed–Frost type of models were used to estimate household and community transmission parameters from household final size data, assuming a constant probability of infection from the community [4–6]. Ball *et al.* [7] generalized this to the so-called ‘households model’ with two levels of mixing, assuming random mixing within households (local) and in the entire population (global), the latter typically at a much lower rate. The analytical tractability of the households model allowed for the theoretical study of epidemic phenomena. This research has led to the definition of threshold parameters such as the reproduction number R*, representing the average number of households infected by a typical infected household in a totally susceptible population [7,8]. Meyers *et al.* [9] used a contact network model in an urban setting incorporating households as complete networks (cliques) to explain the early epidemiology of SARS. Individual-based simulation models of infectious disease transmission incorporate detailed individual-level information in order to account for heterogeneities relevant to the application (e.g. demography, socioeconomics or genetics [10–12]). These models allow for incorporating more detailed structure in specific settings such as schools and workplaces, but typically assume random mixing in households. Studies that particularly highlight within-household transmission and control policies targeting households can be found in [1,13].

Until now, there has been no direct empirical evidence to support the assumption of homogeneous mixing within households. Egocentric contact surveys entailed partially observed within-household contact networks and only allowed for indirect inference of the unobserved network links [14,15]. It has been argued that greater realism could be gained by considering different household compositions and contact heterogeneity within households [16].

In this paper, we describe the first social contact survey specifically designed to study contact networks within households. This study enables us to empirically assess the assumption of homogeneous mixing (e.g. by studying the effects of age and gender on social distance within households). Furthermore, it provides an answer to one of the key questions regarding inference based on household models: how does the density of the contact network scale with the household size [16]? When ignoring contact heterogeneity between household members, the contact network density equals the contact rate between two individuals in a household and is a determinant for the within-household transmission rate of airborne infectious diseases [17,18]. Finally, this study makes it possible to assess reporting quality for diary-reported contact surveys by looking at reciprocity (i.e. symmetry in contact reporting). We use exponential-family random graph models (ERGMs [19]) to develop a plausible model for within-household contact networks and to gain insight into the factors driving contacts between household members. We then compare these empirically grounded ERGMs to the assumption of random mixing using stochastic simulations of an epidemic in the *mise en scene* of the households model with two levels of mixing.

## 2. Results

### (a) Household contact survey

From 2010 to 2011, a survey was conducted to study social contact behaviour in households with young children in Belgium (Flanders and Brussels). A larger similarly designed parallel contact survey of individuals from separate households is described elsewhere [20,21]. Participants were recruited via random-digit dialling, and stratified sampling ensured the representativeness in terms of geographical spread, day and week/weekend distribution and age and gender of the youngest child. All participants were asked to anonymously complete a paper diary recording their contacts during one randomly assigned day without changing their usual behaviour.

The survey focused on households with at least one child of age 12 years or less. Upon sampling, all persons living more than 50% of the time in the household were defined as household members and recruited to take part in the survey. Participants had to record all persons they made contact with during a 24 h period assigned to them. A contact was defined as a two-way conversation at less than 3 m distance or a physical contact involving skin-to-skin touching (either with or without conversation). The information recorded included the exact or estimated age (interval) and gender of each contacted person, physical touching (yes/no), location, frequency and total duration of the contact, in addition to whether the contacted person was a household member. If they contacted someone multiple times on a day, participants specified this as a single contact, along with the estimated contact duration accumulated over the day and set the location category to ‘multiple’ if that person was contacted at two or more different locations.

From the 342 households that participated in the survey, 24 households were excluded because of missing data. We analysed data from 318 households, including 1266 participants who recorded 19 685 contacts in total, with household sizes ranging from 2 to 7. Within-household contacts were identified and matched with other household members using the procedure described in the electronic supplementary material, text. This entailed 3821 identified within-household contacts with 98% reciprocity, indicating a good quality of reporting, as expected in this household setting [22]. We assumed all social contacts to be reciprocal, depicting each household as an undirected network in which nodes represent household members and edges represent contacts within the household. This process resulted in a total of 1946 distinct within-household contacts, of which 1861 (96%) involved physical contact (electronic supplementary material, figure S1).

**Figure 1.**
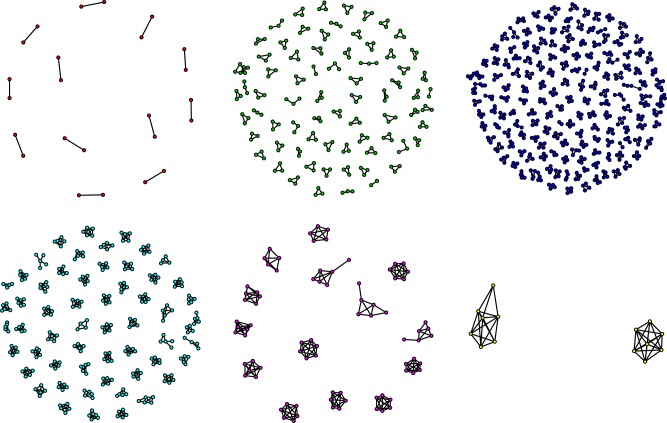
Observed within-household physical contact networks by household size (2 to 7). Nodes represent household members and edges represent physical contacts. (Online version in colour.)

Electronic supplementary material, figure S1 shows that contacts between household members were of long duration, which is consistent with findings from previous social contact surveys [18] and from individual-based simulation models creating so-called synthetic populations [23]. Further, interactions between household members occurred (almost) daily and 66% of household members only met each other at home on their assigned day, whereas 33% met at multiple locations, of which 98% included home. In the following, we focus on physical contacts (with and without conversation) since it has been shown that these better explain the observed age-specific seroprevalence of airborne infections, such as varicella and parvovirus B19, compared to non-physical contacts [24–26]. Figure 1 allows one to appreciate at a glance the diversity in household size and network configurations that we studied through the survey.

Age, gender and household size were used to assign the role of child, mother and father to each household member. Two households were excluded from further analysis due to assignment issues associated with a grandparent and a same-sex couple. The final dataset thus consists of 316 households, including 1259 participants.

Table 1 summarizes the proportion of complete (i.e. fully connected) networks and the mean network density for the within-household physical contact networks by household size, distinguishing week from weekend days and regular from holiday periods. The network density is defined as the ratio of the number of observed edges to the number of potential edges.

**Table 1.** Proportion of complete networks and mean network density, stratified by household (HH) size, for the observed within-household physical contact networks, comparing weekdays and weekend days (top) and regular and holiday periods (bottom).

	weekday			weekend		
HH size	no. HHs	proportion complete	mean density	no. HHs	proportion complete	mean density
2	9	1.00	1.00	3	1.00	1.00
3	53	0.91	0.96	19	0.74	0.88
4	111	0.77	0.93	48	0.85	0.96
5	39	0.64	0.90	18	0.78	0.95
≥6	13	0.46	0.85	3	1.00	1.00
total	225	0.77	0.93	91	0.82	0.94

	regular period			holiday period		
HH size	no. HHs	proportion complete	mean density	no. HHs	proportion complete	mean density
2	9	1.00	1.00	3	1.00	1.00
3	42	0.86	0.94	30	0.87	0.93
4	105	0.82	0.94	54	0.76	0.93
5	38	0.66	0.91	19	0.74	0.92
≥6	12	0.50	0.84	4	0.75	0.98
total	206	0.79	0.93	110	0.79	0.93

Overall, the type of day does not have a large impact on the contacts within households; however, the data suggest some decreasing connectedness with increasing household size, mainly on weekdays and during regular periods. For households of size 4, the observed proportion of complete networks is 0.77 on weekdays and 0.85 on weekend days. Various measures of within-household clustering are defined in the electronic supplementary material, text, and table S1 presents the high degree of physical contact clustering observed within households.

### (b) Modelling within-household physical contact networks

We use ERGMs [19] to model the within-household physical contact networks. We explore the effect of relationships (i.e. contacts between siblings, among children and their parents and between partners), gender-preferential contacts and age effects in children, and the effect of household size, distinguishing small (less than or equal to 3 members), medium (4 members) and large (greater than equal to 5 members) households (electronic supplementary material, table S2). We also explore the presence of higher-order dependency effects between members of the same household, such as clustering (see electronic supplementary material, table S1), by including in the model the number of isolate individuals, 2-stars, triangles and triangles in households of size greater than or equal to 6. A 2-star is a person connected to two other household members and a triangle is a set of three household members such that all three are connected to each other.

The within-household physical contact networks were modelled separately for weekdays and weekends, and the final ERGMs are presented in table 2. Reference categories are child–child contacts (both of age zero) and contacts within households of size 4. The estimates reported in this table are log odds ratios and hence need to be exponentiated to obtain odds ratios. Note that the edge effect is estimated as negative to counterbalance the large within-household edge effect, which is needed because our data do not include between-household contacts. By design, this entails highly significant *p*-values associated with the edges and within-household edges terms (not shown). For both types of days, the effects of gender-preferential contacts and the number of isolates were found to be non-significant (likelihood ratio test *p* = 0.5766 for weekdays). For weekend days, no significant effect of household size was found, and the model was further reduced to an 8-parameter model (likelihood ratio test *p* = 0.5134). On weekdays, the odds of a physical contact occurring in a household of size less than or equal to 3 and greater than or equal to 5 are estimated to be 2.10 and 0.67 times the odds of a physical contact occurring in a household of size 4, respectively. Thus, the network density for physical contacts decreases with increasing household size. Further, on both types of days, the probability for siblings to make physical contact decreases with increasing age. This result implies that only the odds of a physical contact between older siblings are less than between father and child. Electronic supplementary material, figure S3 illustrates this age threshold in both the weekday and weekend day models. For households of size less than or equal to 5, the odds of a physical contact that will complete a triangle are estimated to be 7.85 and 35.87 times the odds of a physical contact that will not complete a triangle on weekdays and weekend days, respectively. This results demonstrates the overall high degree of contact clustering within households. On weekdays, the degree of clustering is slightly lower in households of size ≥6 (conditional odds of 5.93).

**Table 2.** ERGM for within-household physical contact networks on week and weekend days: parameter estimates and Wald test *p*-values, log-likelihood and AIC.

	weekday		weekend	
network statistic	estimate	*p*-value	estimate	*p*-value
edges	−28.16		−20.63	
within-household edges	28.97		22.78	
child–father edges	−0.60	0.23	−1.15	0.45
child–mother edges	0.16	0.76	0.14	0.93
father–mother edges	0.27	0.66	−0.76	0.63
age effect children	−0.07	<0.01	−0.18	<0.01
small households (≤3)	0.74	<0.01		
large households (≥5)	−0.40	<0.01		
2-stars	−0.26	0.25	−0.87	0.01
triangles	2.06	<0.01	3.58	<0.01
triangles in households ≥6	−0.28	0.02		
log-likelihood	−306.80		−65.98	
AIC	635.59		147.95	

The goodness-of-fit of the models is assessed by simulating new sets of physical contact networks from the fitted ERGM and by comparing specific contact network characteristics that are not included in the model to the observed ones. We compare the proportion of complete networks, the mean network density and the proportion of observed versus potential triangles by household size. Overall, the final ERGMs fit the data well, as indicated in electronic supplementary material, tables S3–S6 and figures S4–S6.

### (c) Epidemic spread in a community of households

We simulate the spread of a newly emerging infection in a closed fully susceptible population of households using a discrete-time chain binomial SIR (susceptible–infected–recovered) model [27]. The 225 households from the contact survey that were analysed using the weekday ERGM are used to construct the community of households. We assume two levels of mixing similar to the households model in [7]: high-intensity mixing within households and low-intensity ‘background’ random mixing in the community (i.e. between households). Two different configurations for within-household mixing are compared: random mixing and empirical-based mixing, where the latter refers to physical contact networks simulated from the fitted ERGMs. For each epidemic simulation, two sets of within-household contact networks are drawn from the ERGMs, one for time points defined as weekdays and one for time points defined as weekend days. These weekday and weekend contacts are kept fixed during the entire simulation.

Since we aim to study the effect of contact heterogeneity, we assume that susceptibility and infectiousness are invariant with age. Further, we assume that there is no latent period (i.e. individuals are infectious immediately upon acquiring infection). At each time step (including time of infection), infected individuals recover with a constant probability of 0.22, resulting in a mean infectious period of approximately 3.5 days. The values for the transmission parameters are chosen in line with literature estimates for influenza based on household final size and symptom onset data (electronic supplementary material, table S7). The first day of the epidemic is randomly determined to be a week or weekend day and is started by infecting three random individuals. The epidemic is then tracked until all infected individuals are recovered and no new infections have occurred. The results are presented as the means over simulations with 95% percentile intervals indicated between square brackets. The box plots include lower and upper hinges that correspond to the first and third quartiles. The whiskers extend from the hinges to the smallest/largest values no further than 1.5 times the interquartile range (IQR). Outlying points are plotted individually. The notches extend the median by $1.58\times IQR/\sqrt{n}$. In the figures, small outbreaks, defined as outbreaks with a final size of <100 individuals that lasted less than 60 days, are excluded from display.

#### (i) Scenario 1

The results obtained from 1000 stochastic epidemic simulations are shown in the electronic supplementary material, figures S7–S10. The proportion of small outbreaks is significantly smaller in the random mixing setting compared to empirical-based mixing, 0.43 and 0.50, respectively (Fisher’s exact test, *p*-value < 0.01). The mean proportion of individuals ultimately infected and the mean proportion of households infected are slightly greater under random mixing: 0.39 [0.12, 0.56] versus 0.36 [0.12, 0.53] (Wilcoxon rank sum test, *p*-value < 0.01), and 0.70 [0.28, 0.88] versus 0.67 [0.29, 0.86] (*p*-value < 0.01), respectively (electronic supplementary material, figure S10). Furthermore, the household attack rate, defined as the mean proportion of individuals infected per household [4], increases with household size under both settings (electronic supplementary material, figure S8).

#### (ii) Scenario 2

**Figure 2.**
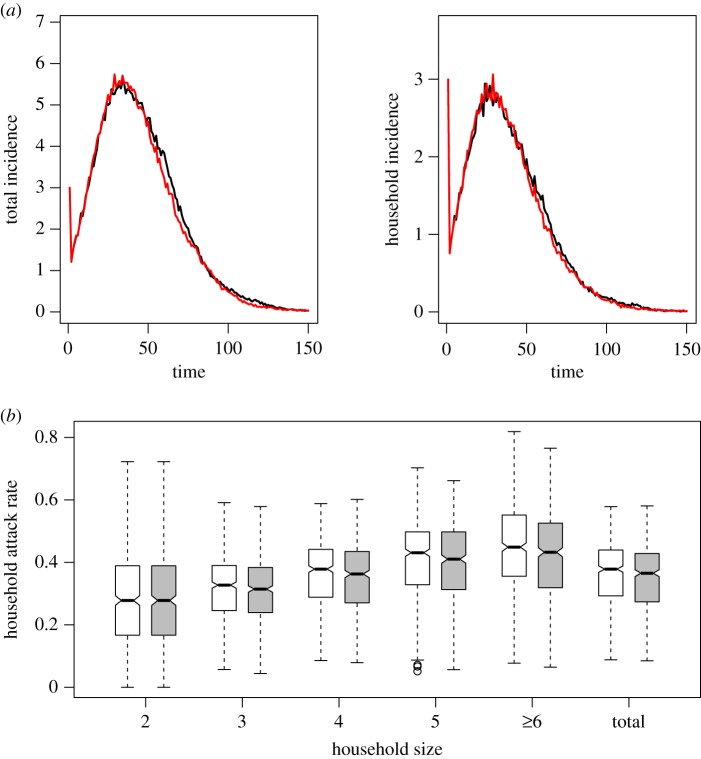
(*a*) Mean infection incidence over time at the individual (left; number of newly infected individuals over time) and household (right; number of newly infected households over time) levels assuming random (black) and empirical-based mixing (red) within households including a density scaling factor. (*b*) Household attack rates (mean proportion of infected individuals per household) by household size assuming random (white) and empirical-based mixing (grey) within households including a density scaling factor.

In scenario 1, the small differences between the network model and the random mixing scenario could be due simply to different densities rather than to any particular characteristic of the network structure. In this setting, we calibrate in order to make a fairer comparison between the two scenarios (see electronic supplementary material, text). Figure 2*a*,*b* and electronic supplementary material, figures S11 and S12 present the results obtained from 1000 simulations. Figure 2*a* shows the same epidemic dynamics over time, and figure 2*b* shows that the relation between household attack rate and household size is the same in both settings. Furthermore, there are no significant differences in the mean final fraction of individuals (0.37 [0.13, 0.52] versus 0.36 [0.12, 0.53]; *p*-value 0.11) and mean final fraction of households (0.68 [0.31, 0.86] versus 0.67 [0.29, 0.86]; *p*-value 0.19; electronic supplementary material, figure S12) for random mixing compared to empirical-based mixing. The proportion of small outbreaks is similar in both settings, 0.48 and 0.50 (Fisher’s exact test, *p*-value 0.40).

A more ‘extreme’ setting, focusing on physical contacts with a duration of more than 4 h and assuming a higher within-household transmission rate, yields a lower incidence for empirical-based mixing regardless of correcting for the within-household density (see electronic supplementary material, figures S13–S16).

## 3. Methods

Let ***Y*** denote the random adjacency matrix of an undirected network, where *Y*_*ij*_ = *Y*_*ji*_ = 1 if person *i* and *j* made physical contact and zero if not, and let *Ω* denote the support of ***Y*** (i.e. the set of all obtainable networks). In an ERGM, the probability of observing a set of network edges is defined as follows:

$$P_{\theta ,\Omega }(Y=y)=\frac{\exp\{\theta^{T}g(y,X)\}}{\kappa (\theta ,\Omega )},\;y\in \Omega ,$$

where ***g***(***y***, ***X***) is a vector of network statistics that may depend on additional covariate information ***X***, with ***θ*** the corresponding vector of coefficients, and *κ*(***θ***, *Ω*) is a normalizing factor. By using an alternative model specification (see SI text), ***θ*** can be interpreted as the increase in the conditional log-odds of the network, per unit increase in the corresponding component of ***g***(***y***, ***X***), resulting from switching a specific *Y*_*ij*_ from 0 to 1 while leaving the rest of the network fixed at $Y_{ij}^{c}$.

We infer the processes driving physical contacts between household members by incorporating network statistics based on nodal covariate information (see electronic supplementary material, table S2). Although our analysis is focused on within-household contact networks, we fitted a single ERGM including all households. We include in our model a household effect that captures the tendency to contact others in one’s own household. Because there are no between-household contact reports present in our survey, the coefficient for the household preference effect should be estimated to be extremely large; thus, the probability of between-household contact is essentially zero.

Approximate maximum-likelihood estimates are obtained using a stochastic Markov chain Monte Carlo (MCMC) algorithm [28]. MCMC estimation is performed with the ‘ergm’ package in R [29,30] that is part of the ‘statnet’ suite of packages for statistical network analysis [31–33].

More detail can be found in the electronic supplementary material.

## 4. Discussion

In this paper, we presented the first social contact study focusing specifically on contact networks within households. The inference of within-household contact networks in previous studies was based on egocentric contact data from the POLYMOD study [14,15,18] or on data less representative of the general population with limited sample sizes (rural Peru and Kenya [34,35]). Estimates of the proportion of complete networks inferred by Potter *et al*. [14,15] ranged from 0.34 to 0.65 for households of size 4 and are thus less than the proportions that we observed (0.77 on weekdays and 0.85 on weekend days). The former estimates were based on partially observed within-household contact networks and therefore likely underestimated the true proportion of complete networks. For the purpose of studying household contacts, the current household-based survey design is considered an improvement on the individual-based survey design (POLYMOD study [18]).

We analysed the household network data using ERGMs to assess the effect of factors such as role in the household, gender, children’s age and household size on physical contacts within households. We found that contacts between father and children are less likely than contacts between father and mother, between mother and children and between siblings (except older siblings). These results are in line with conclusions obtained by de Greeff *et al.* [36]. They analysed data regarding pertussis in households with young infants and found that fathers were less likely to contract pertussis than other household members. The targeted vaccination of mothers and siblings was found to be most effective, as siblings were more likely to introduce an infection into the household. The result that children are more likely to transmit an infection than adults in the same household was also found for influenza [3], and our study shows that contact heterogeneity could play a role here. Hence, the specific contact patterns characterized by the ERGMs in this paper could prove useful for agent-based modelling of infectious disease spread. Further, in most household transmission models, it is assumed that the mean contact degree is proportional to *z*^*w*^, where *z* is household size and *w* controls the extent of the density dependence. We found that the contact density decreases and that the mean number of contacts increases with increasing household size (see table 1, electronic supplementary material S1), which implies that *w* has to be contained in the open interval ]0, 1[. This result supplements findings from studies based on household epidemic data regarding close-contact infections [3,36,37] from a social contact data perspective. Finally, by simulating epidemics in a two-level SIR setting using literature-based influenza parameters, we found that solely incorporating contact heterogeneity has no impact on epidemic spread. This result indicates that in this setting the assumption of random mixing between household members may be an adequate approximation of social contact behaviour for infections transmitted via close contacts. However, the results do suggest that accounting for the within-household contact density is important. This result is well established for pathogens, such as influenza, that are transmitted via casual interactions (see, for example, Bansal *et al.* [38]). Furthermore, we found that in a more extreme setting with intenser contacts and a higher within-household transmission rate, a density correction is insufficient to bridge the differences between both mixing assumptions. This result suggests that informing mixing between household members with social contact data could impact modelling efforts in certain settings.

Our study has some limitations and assumptions. We assume that a contact occurred if it was reported by at least one household member. Thus, contacts forgotten by both members could result in an underestimation of the network density. Potter *et al.* [39] developed a model address the issue of reporting error affecting network edges. However, given that the high reciprocity rates (98%) indicate a very good reporting quality of the survey, we believe that such an adjustment will not have a significant impact on our conclusions. Further, our results depend on the contact definition used to determine the within-household network links and cannot be generalized to the spread of any infectious disease. Based on exploration of various contact definitions when using POLYMOD contact data to estimate age-specific varicella transmission rates [25], we opted to use physical contacts in this study as a surrogate of potential transmission events for close-contact infections, such as influenza and smallpox, although even for two airborne infections, different networks may be appropriate because differing levels of interaction will be required to constitute an effective contact [40]. Additionally, the contact survey only included households with at least one child of age 12 years or less. This subgroup was considered to be most relevant as this group is mostly affected by increased exposure to airborne/droplet infections due to out-of-home care and school attendance [41]. Children older than 12 years are less at risk because of prior immunity and better hygiene. Therefore, contacts within households with young children are considered the most important drivers for transmission. Finally, even though a week/weekend distinction was made, static networks were used to simulate epidemic spread. This simplified approach fails to capture that missing contacts are likely not consistently missing but rather a snapshot of a particular day.

The methods in this paper could be extended in a number of manners, which would be interesting topics of future research. We observed a relationship-specific heterogeneity in duration of contact (presented in electronic supplementary material, figure S2) and an impact of this duration on epidemic spread, which might be relevant for some diseases. The ERGM framework can be adapted to a ‘valued within-household contact networks’ model [42], with the value of a contact determined by its total duration, and by weighting the transmission rates in the epidemic simulation model accordingly. It is also of potential interest to capture the temporal dynamics of within-household contacts and to simulate the impact of contact formation and dissolution on the spread of infection [43,44]. Combining time-use data with social contact data would allow for inferring the potential timing of contacts with household members and to estimate dynamic within-household contact networks. This combination would also be valuable to inform large-scale individual-based simulation models of infectious disease spread. Further, the exploration of potential differences in the distribution of the generation interval in a random-mixing setting versus empirical-based mixing is the topic of current research. Finally, combining the model for within-household contact networks developed in this paper with epidemic data from a similar community of households would allow for improving the estimates of age-specific heterogeneity in susceptibility and infectiousness for infections such as influenza [6].

This study provides unique insights into within-household contacts, which are considered to be important drivers of many close-contact infections. It presents the first empirical evidence resulting from a large household contact survey supporting the use of the random mixing assumption in epidemic models incorporating household structure.

## Supplementary Material

Supplementary Information

## Data accessibility

The datasets and R code supporting this article can be accessed via http://www.simid.be/hhpapercodeanddata/.
