# Supplementary material for: Household members do not contact each other at random: implications for infectious disease modelling
Source: Proc Biol Sci. 2018 Dec 12;285(1893):20182201. doi: 10.1098/rspb.2018.2201 (PMC6304037; doi:10.1098/rspb.2018.2201)
Supplement: Supplementary Information [file rspb20182201supp1.pdf]

Supporting Information - Household Members Do Not Contact Each Other at Random: Implications for Infectious Disease Modelling

Nele Goeyvaerts[a,1], Eva Santermans[a,1,2], Gail Potter[b], Andrea Torneri[c], Kim Van Kerckhove[a], Lander Willem[c], Marc Aerts[a], Philippe Beutels[c], and Niel Hens[a,c]

[a]Interuniversity Institute for Biostatistics and statistical Bioinformatics, UHasselt, Belgium
[b]The Emmes Corporation, Rockville MD, USA
[c]Centre for Health Economics Research and Modelling Infectious Diseases, Vaccine & Infectious Disease Institute, University of Antwerp, Belgium
[1] Current affiliation: Janssen Research & Development, Beerse, Belgium
[2] To whom correspondence should be addressed. E-mail: eva.santermans@uhasselt.be

1 Household contact survey

In 2010-2011, a survey was conducted to study social contact behavior in households with young children in Belgium (Flanders and Brussels). A larger similarly designed parallel contact survey in individuals from separate households is described elsewhere (Willem et al., 2012; Kifle et al., 2015). Participants were recruited by random digit dialing and stratified sampling ensured representativeness in terms of geographical spread, day and week-weekend distribution, and age and gender of the youngest child. All participants were asked to anonymously complete a paper diary recording their contacts during one randomly assigned day without changing their usual behavior.

Two types of contact diaries were used, adapted to the age of the participants: one for children (0-12 years) designed to be filled by a proxy, and one for adolescents and adults ($>$ 12 years). The diaries were sent and collected by mail. Participants were reminded by phone to fill in the diary one day in advance and followed up the day after. Data were single entered in a computer database and independently checked.

Participants had to specify whether or not the contacted person belonged to their household. Contacts reported as household contacts are linked to other household members using the following criteria: matching household identification number, gender and age (allowing the recorded age to deviate from the true age by 1 year). As such, all contacts reported as household contacts could be linked to a unique household member. Amongst the remaining contacts, i.e. with missing or negative household member indicator, that occurred at home, an additional small subset of household contacts is identified using the same criteria as before but requiring an exact age match. From the 19685 contacts reported in total, 3821 (19%) are identified as within-household contacts.

From these 3821 within-household contacts, 98% are reciprocal of which 96% with equal touching indicator, 97% with equal frequency and 84% with equal duration. Social contacts have to be reciprocal thus we analyze undirected within-household contact networks. Non-reciprocal household contacts in the data set were assigned to reporting issues and imputed. Contact characteristics of reciprocal contacts are merged such that the most intense contact value is retained and the location category is set to ‘multiple’ if two or more different locations are reported. This results in a total of 1946 distinct within-household

contacts including 1861 physical contacts. There are 9 participants who did not record any contact with other household members and are referred to as isolates.

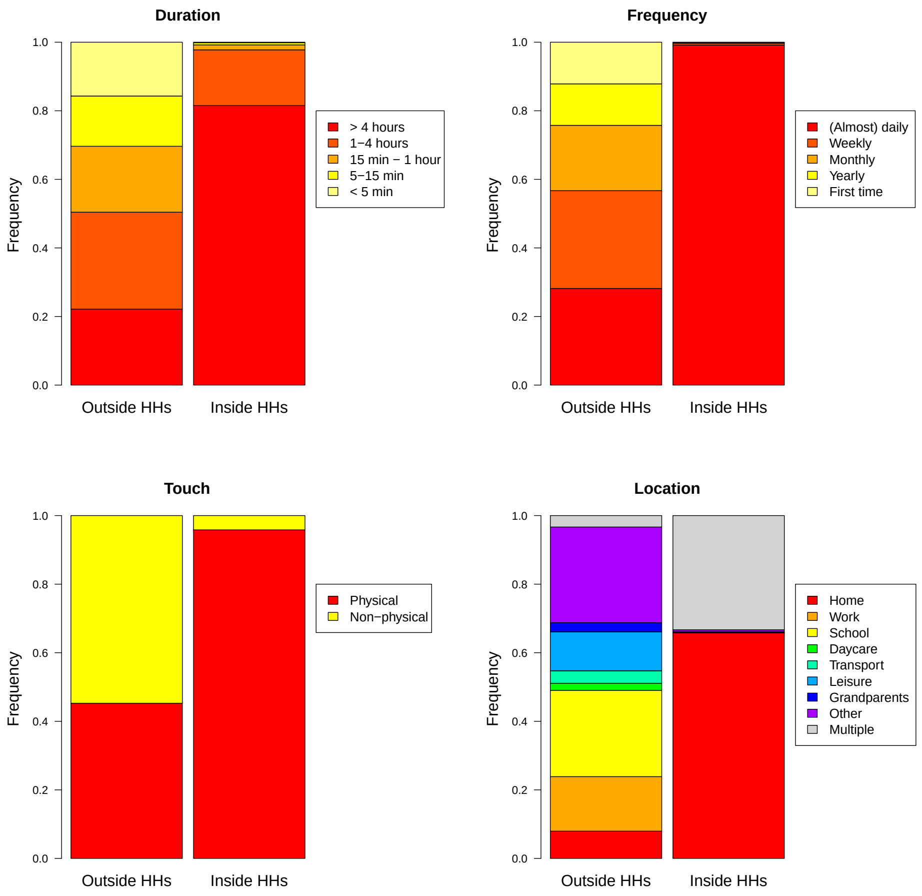

Figure S1: Distribution of contact intensity (duration, frequency and touching) and location for all record contacts with non-household (left) and household members (right).

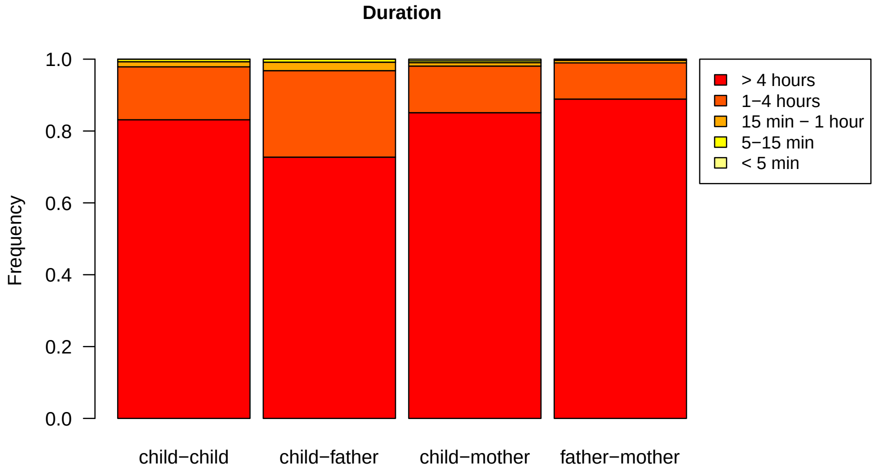

Figure S2: Distribution of within-household contact duration by type of relationship, including both physical and non-physical contacts.

2 Modeling within-household physical contact networks

Clustering

We consider various measures of within-household clustering: the clustering coefficient (Kolaczyk, 2009), the mean correlation coefficient (Morris et al., 2008) and the proportion of observed versus potential triangles, defined as:

$$\begin{aligned}\text{Clustering coeffcient} &= \frac{3 \cdot \#\text{triangles}}{\#\text{connected triples}};\\ \text{Mean correlation coeffcient} &= \frac{\#\text{triangles}}{\#\text{triangles} + \#\text{2-stars} \notin \text{triangle}};\\ \text{Proportion observed vs. potential triangles} &= \frac{\#\text{triangles}}{\sum_h \binom{\text{size}(h)}{3}}.\end{aligned}$$

Table S1: Observed physical contact networks: average degree and various measures of within-household clustering, stratified by household size.

HH size	Nr. HHs	Average degree	Clustering coefficient	Mean correlation coefficient	Proportion observed vs. potential triangles
2	12	1.00	NA	NA	NA
3	72	1.88	0.96	0.90	0.86
4	159	2.81	0.97	0.91	0.87
5	57	3.66	0.96	0.90	0.83
≥ 6	16	4.51	0.96	0.88	0.77
Total	316	2.94	0.96	0.90	0.83

Exponential random graph model

The following model specification clarifies the interpretation of $\boldsymbol{\theta}$ (Hunter et al., 2008). For a specific pair of nodes (i, j), define the vector of change statistics as follows:

$$\boldsymbol{\delta_g}(\boldsymbol{y}, \boldsymbol{X})_{ij} = \boldsymbol{g}(\boldsymbol{y}_{ij}^{+}, \boldsymbol{X}) - \boldsymbol{g}(\boldsymbol{y}_{ij}^{-}, \boldsymbol{X}),$$

where $\boldsymbol{y}_{ij}^{+}$ and $\boldsymbol{y}_{ij}^{-}$ are the networks realized by fixing $y_{ij} = 1$ and $y_{ij} = 0$, respectively, while leaving all the rest of $\boldsymbol{y}$ fixed. This allows for a logistic interpretation of the coefficients in $\boldsymbol{\theta}$:

$$\text{logit}\{P_{\boldsymbol{\theta},\Omega}(Y_{ij} = 1|\boldsymbol{Y}_{ij}^{c} = \boldsymbol{y}_{ij}^{c})\} = \boldsymbol{\theta}^{T}\boldsymbol{\delta}_{\boldsymbol{g}}(\boldsymbol{y}, \boldsymbol{X})_{ij}, \tag{1}$$

where $\boldsymbol{Y}_{ij}^{c}$ represents the rest of the network other than Y_{ij}. Thus, $\boldsymbol{\theta}$ reflects the increase in the conditional log-odds of the network, per unit increase in the corresponding component of $\boldsymbol{g}(\boldsymbol{y}, \boldsymbol{X})$, resulting from switching a particular dyad Y_{ij} from 0 to 1 while leaving the rest of the network fixed at $\boldsymbol{Y}_{ij}^{c}$.

The following network statistics are considered in the ERGMs.

Table S2: Network statistics considered in the ERGMs, where an edge is defined as a physical contact between two individuals. Reference categories are child-child mixing, boy-girl mixing, and mixing within households of size 4.

Network statistic	Legend
Edges	Total number of edges
Within-household edges	Total number of edges within households
Child-father edges	Total number of edges between children and fathers
Child-mother edges	Total number of edges between children and mothers
Father-mother edges	Total number of edges between partners
Boy-boy edges	Total number of edges between male children
Girl-girl edges	Total number of edges between female children
Age effect children	The sum of age(i) and age(j) for all edges (i, j) between siblings
Small (<=3) households	Total number of edges within households of size ≤ 3
Large (>=5) households	Total number of edges within households of size ≥ 5
Isolates	Total number of isolates
2-stars	Total number of 2-stars
Triangles	Total number of triangles
Triangles in households of size ≥ 6	Total number of triangles in households of size ≥ 6

Both the total number of edges (physical contacts) and the total number of edges within households are included in the ERGM. Constrained optimization with fixed coefficients for these statistics as proposed by Potter and Handcock (2010) does not entail a plausible approximation of the likelihood for our data. Therefore, we use unconstrained optimization and check whether the probability of physical contact between non-household members is approximately zero.

The triangle term estimates a transitivity effect, i.e. the increase in log odds of contact between two people due to the fact that they have a third contact in common. Inclusion of triangle terms in ERGM models has been found to lead to "model degeneracy" in some cases (Handcock, 2003). Model degeneracy occurs when the maximum likelihood estimate places most probability on a small set of possible networks (e.g., all mass on the complete network). It results from the fact that the triangle term does not impose decreasing marginal returns on the number of mutual contacts made by the two individuals in question. For example, the increase in log odds of contact between a pair whose number of mutual contacts increases from zero to one is forced to be the same as the increase in log odds of contact between a pair whose number of mutual contacts increases from ten to eleven. Alternate ERGM terms have been proposed which instead, more realistically, model decreasing marginal returns of the number of mutual contacts on the log odds of contact (Hunter, 2007). However, model degeneracy was not found to be a problem in our case, possibly because the unique structure of our data set, which includes a large number of households but includes no between-household contacts, prevents an "avalanche effect" of triangles towards the complete network.

Approximate maximum likelihood estimates are obtained using a stochastic Markov Chain Monte Carlo (MCMC) algorithm (Geyer and Thompson, 1992). In short, a distribution of random networks is simulated from a starting set of parameter values using MCMC and the parameter values are refined by

comparing this distribution of networks against the observed network in a Newton-Raphson type algorithm, repeating this process until the parameter estimates stabilize (Robins et al., 2007). We use a burn-in of length 10^6, intervals between sampled networks of length 10^3 and a total sample size equal to $5 \cdot 10^5$. The initial value of $\boldsymbol{\theta}$ is obtained by maximum pseudolikelihood estimation, considering equation (1) as a logistic regression model assuming all Y_{ij} mutually independent (Strauss and Ikeda, 1990).

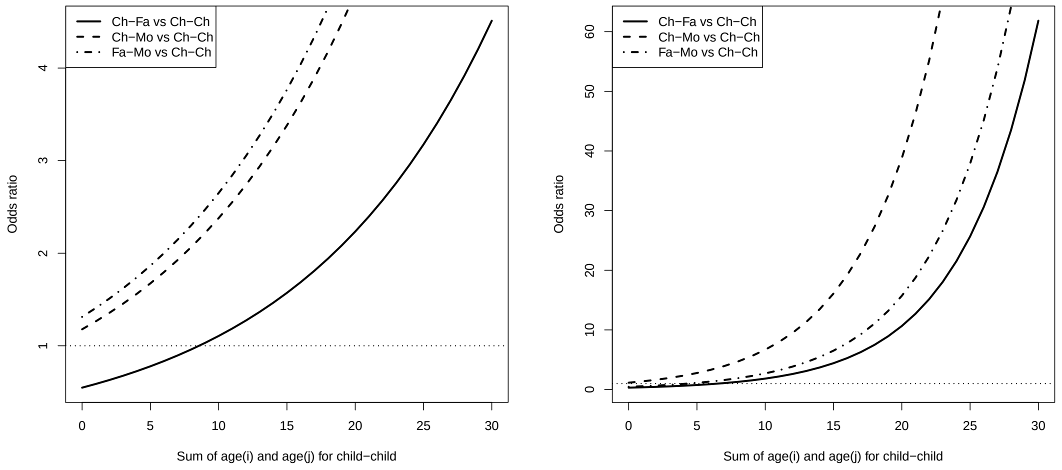

Figure S3: Interpretation of relationship contacts and age effect statistics of the ERGM: ratio of the odds of physical contact occurring between two relatives versus a pair of siblings, as a function of the sum of the siblings' ages. Left: weekday, right: weekend day.

Goodness-of-fit

We simulate 1000 networks using a burn-in of length 10^7 and intervals of length 10^6 between sampled networks. The first simulated Markov chain begins at the initial network and the end of one simulation is used as the start of the next simulation.

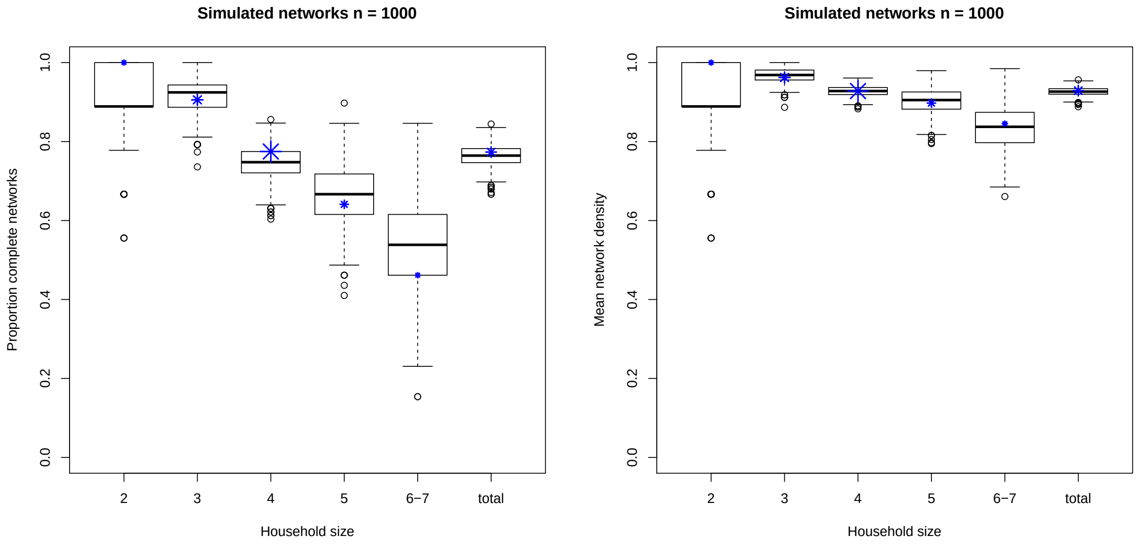

Figure S4: Proportion of complete networks (left) and mean network density (right) on a weekday: distribution of the simulated values from the ERGM for within-household physical contact networks (boxplots) and the observed values (blue stars with size proportional to the sample size). Box plots present lower and upper hinges that correspond to the first and third quartiles. The whiskers extend from the hinges to the smallest/largest values no further than 1.5*IQR. Outlying points are plotted individually.

Table S3: Proportion of complete networks and mean network density, stratified by household size and presented with the median and 95% percentile range of 1000 networks simulated from the ERGM for within-household physical contact networks on a weekday.

HH size	Nr. HHs	Proportion complete				Mean density			
		Observed	Median	Q 2.5%	Q 97.5%	Observed	Median	Q 2.5%	Q 97.5%
2	9	1.00	0.89	0.67	1.00	1.00	0.89	0.67	1.00
3	53	0.91	0.92	0.83	0.98	0.96	0.97	0.93	0.99
4	111	0.77	0.75	0.66	0.82	0.93	0.93	0.90	0.95
5	39	0.64	0.67	0.51	0.79	0.90	0.91	0.84	0.96
≥ 6	13	0.46	0.54	0.31	0.77	0.85	0.84	0.73	0.93
Total	225	0.77	0.76	0.71	0.81	0.93	0.93	0.91	0.95

Table S4: Proportion of observed versus potential triangles, stratified by household size and presented with the median and 95% percentile range of 1000 networks simulated from the ERGM for within-household physical contact networks on a weekday.

HH size	Nr. HHs	Proportion observed vs. potential triangles			
		Observed	Median	Q 2.5%	Q 97.5%
3	53	0.91	0.92	0.83	0.98
4	111	0.85	0.84	0.78	0.89
5	39	0.81	0.82	0.71	0.90
≥ 6	13	0.71	0.71	0.56	0.87
Total	216	0.80	0.80	0.75	0.85

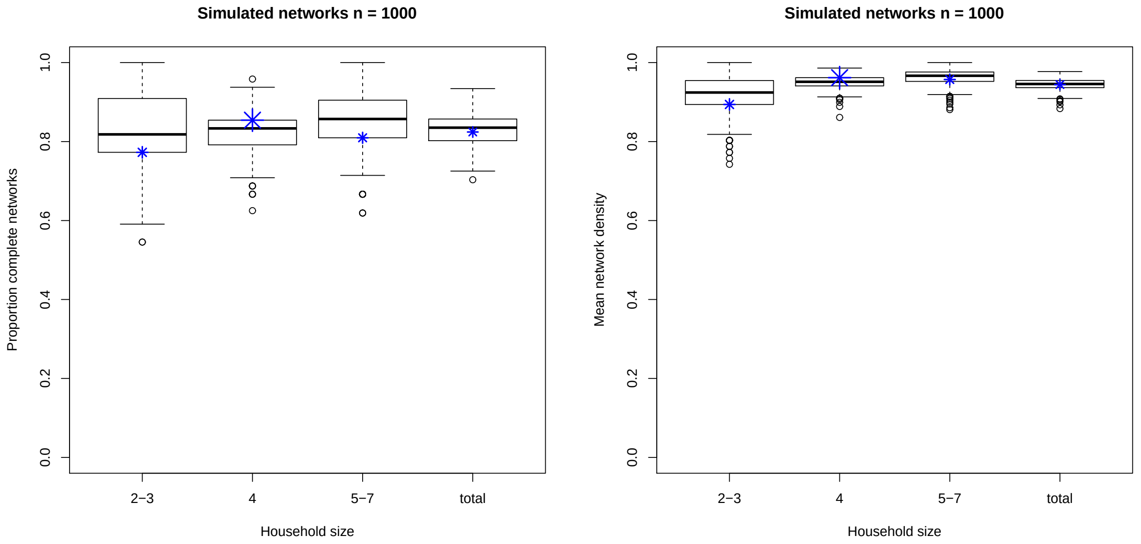

Figure S5: Proportion of complete networks (left) and mean network density (right) on a weekend day: distribution of the simulated values from the ERGM for within-household physical contact networks (boxplots) and the observed values (blue stars with size proportional to the sample size). Box plots present lower and upper hinges that correspond to the first and third quartiles. The whiskers extend from the hinges to the smallest/largest values no further than 1.5*IQR. Outlying points are plotted individually.

Table S5: Observed proportion of complete networks and mean network density, stratified by household size and presented with the median and 95% percentile range of 1000 networks simulated from the ERGM for within-household physical contact networks on a weekend day.

HH size	Nr. HHs	Proportion complete				Mean density			
		Observed	Median	Q 2.5%	Q 97.5%	Observed	Median	Q 2.5%	Q 97.5%
≤ 3	22	0.77	0.82	0.68	0.95	0.89	0.92	0.82	0.98
4	48	0.85	0.83	0.73	0.92	0.96	0.95	0.92	0.98
≥ 5	21	0.81	0.86	0.71	0.95	0.96	0.97	0.92	0.99
Total	91	0.82	0.84	0.76	0.90	0.94	0.95	0.92	0.97

Table S6: Proportion of observed versus potential triangles, stratified by household size and presented with the median and 95% percentile range of 1000 networks simulated from the ERGM for within-household physical contact networks on a weekend day.

HH size	Nr. HHs	Proportion observed vs. potential triangles			
		Observed	Median	Q 2.5%	Q 97.5%
3	19	0.74	0.84	0.63	0.95
4	48	0.91	0.89	0.83	0.94
≥ 5	21	0.93	0.94	0.88	0.98
Total	88	0.91	0.91	0.87	0.95

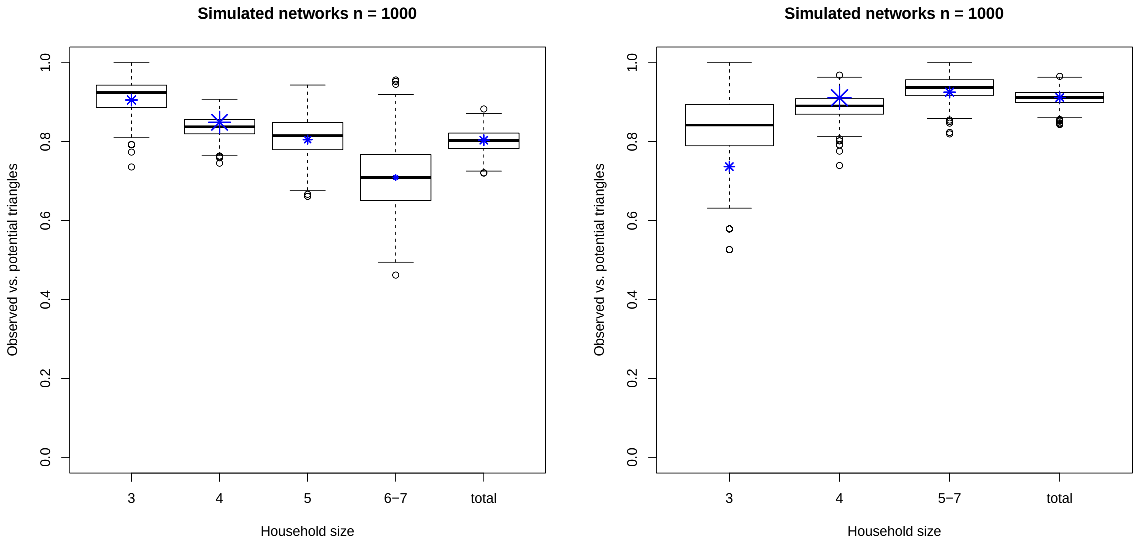

Figure S6: Proportion of observed versus potential triangles on a weekday (left) and on a weekend day (right): distribution of the simulated values from the ERGM for within-household physical contact networks (boxplots) and the observed values (blue stars with size proportional to the sample size). Box plots present lower and upper hinges that correspond to the first and third quartiles. The whiskers extend from the hinges to the smallest/largest values no further than 1.5*IQR. Outlying points are plotted individually.

3 Epidemic simulation model

Scenario 1

The epidemic simulation model is defined as follows: at time step t (in days), assuming infection is spread by means of physical contacts, each susceptible i acquires infection with probability:

$$\begin{aligned} p_{i,1}(t) &= 1-(1-\beta_h)^{\sum_{j\neq i \in h_i} y_{ij} I_j(t)} \cdot (1-\beta_{c,11})^{\sum_{j\notin h_i} I_{j,1}(t)} \cdot (1-\beta_{c,12})^{\sum_{j\notin h_i} I_{j,2}(t)}, \\ p_{i,2}(t) &= 1-(1-\beta_h)^{\sum_{j\neq i \in h_i} y_{ij} I_j(t)} \cdot (1-\beta_{c,21})^{\sum_{j\notin h_i} I_{j,1}(t)} \cdot (1-\beta_{c,22})^{\sum_{j\notin h_i} I_{j,2}(t)}, \end{aligned}$$

where index 1 corresponds to children $\leq$ 18 years and index 2 to adults $>$ 18 years. β_h denotes the within-household transmission probability per physical contact, per time step. The 2×2 community transmission probability matrix $\boldsymbol{\beta_c}$ (with $\beta_{c,12} = \beta_{c,21}$) is taken directly proportional to the per capita physical contact rates estimated from the Belgian POLYMOD contact survey, with a proportionality constant q_c (Mossong et al., 2008; Goeyvaerts et al., 2010).

$$\boldsymbol{\beta_c} = q_c \begin{bmatrix} 17.35 & 6.26 \\ 6.26 & 7.88 \end{bmatrix} \cdot 10^{-7}.$$

Further, y_{ij} denotes the observed adjacency matrix and under the random mixing scenario, y_{ij} equals 1 for all household members i and j. Finally, h_i denotes the household of node i and $I_j(t)$ indicates whether node j is infected (1) or not (0) at time t with subscripts referring to children and adults.

Values β_h and q_c are chosen in line with literature estimates (Table S7): $\beta_h = 0.05$ and $\overline{\boldsymbol{\beta_c}} = 0.00026$, where $\overline{\boldsymbol{\beta_c}}$ is the mean of the elements of $\boldsymbol{\beta_c}$. These parameter values result in estimates of the community probability of infection for children and adults ($\overline{\text{CPI}}_{\text{child}}$ and $\overline{\text{CPI}}_{\text{adult}}$) between 0.18 and 0.20, and 0.11 and 0.12, respectively. Furthermore, the probability to escape infection from an infected household member per day is $q_{HH} = 1 - \beta_h = 0.95$.

Results from 1000 stochastic epidemic simulations are shown below.

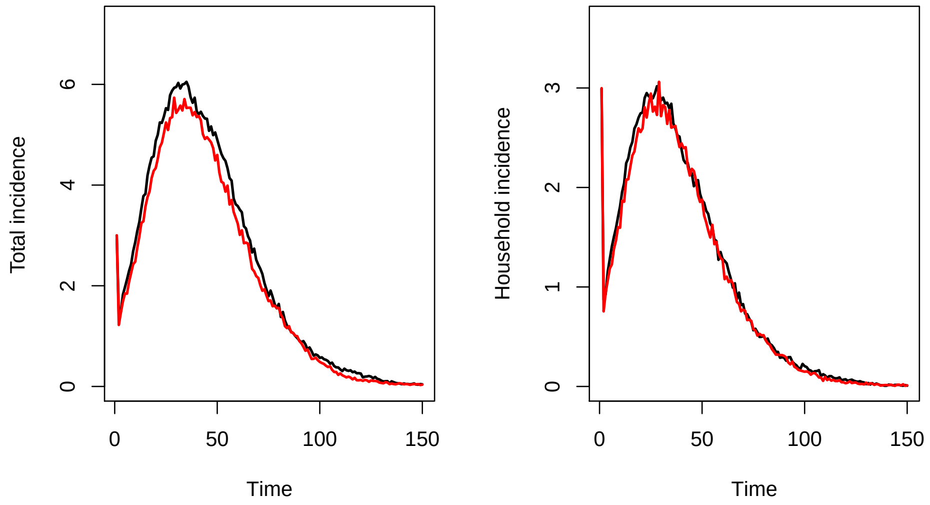

Figure S7: Mean infection incidence over time at the individual (left; number of newly infected individuals over time) and household level (right; number of newly infected households over time) assuming random (black) and empirical-based mixing (red) within households. Small outbreaks are excluded from display.

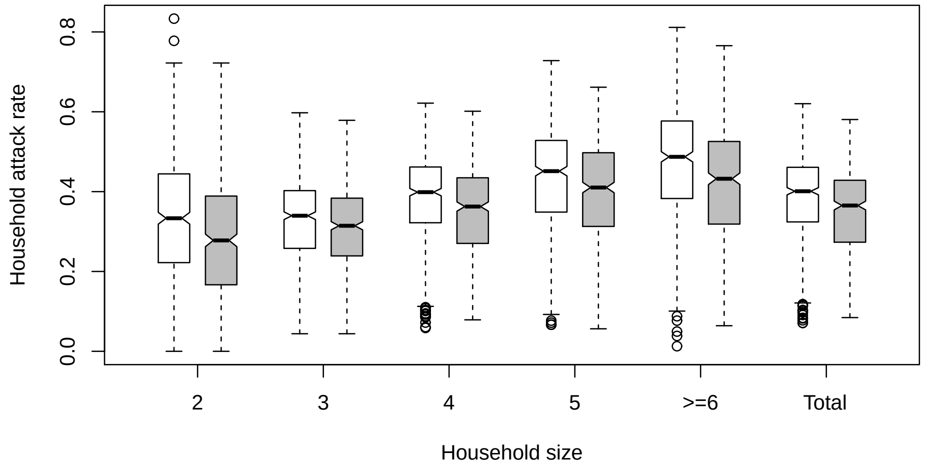

Figure S8: Household attack rates (mean proportion of infected individuals per household) by household size assuming random (white) and empirical-based mixing (gray) within households. Small outbreaks are excluded from display.

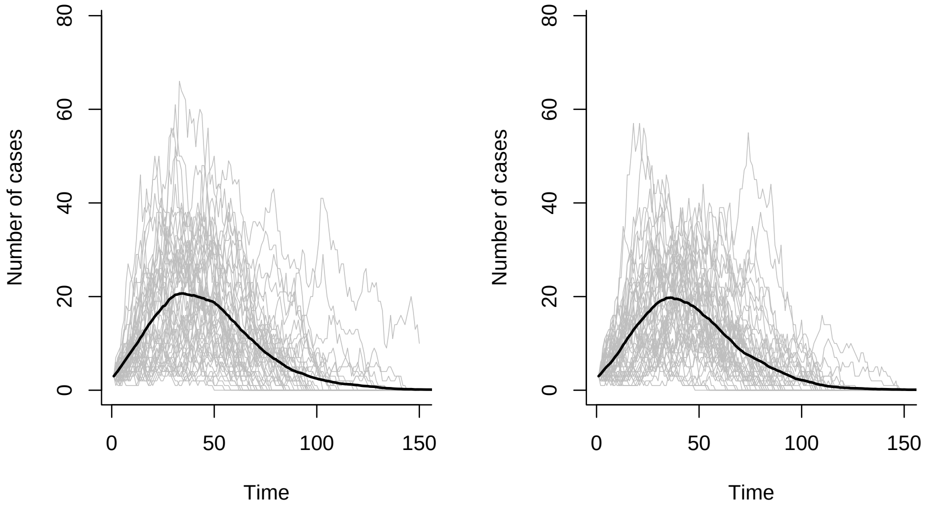

Figure S9: Total number of cases over time assuming random (left) and empirical-based mixing (right) within households. Black solid curves represent the mean and light curves correspond to 50 randomly chosen epidemics. Small outbreaks are excluded from display.

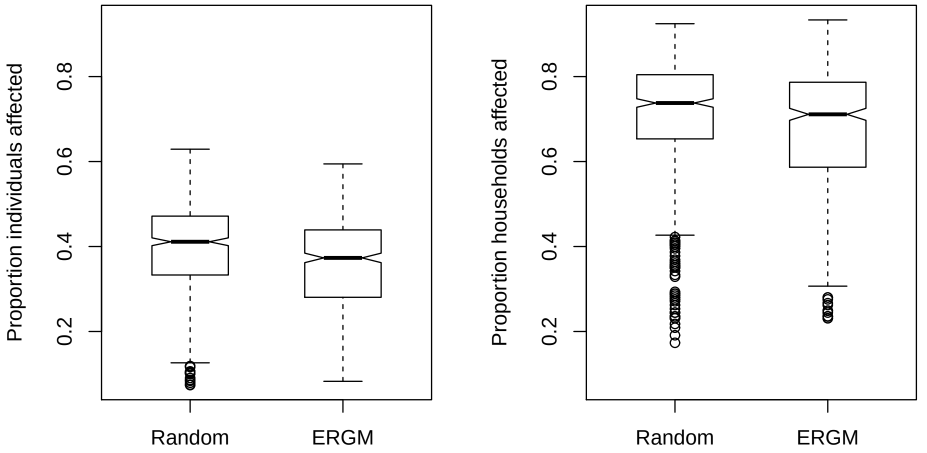

Figure S10: Final fraction of affected individuals (left) and households (right) for 1000 simulations of a stochastic SIR epidemic process on a 2-level households model assuming random and empirical-based mixing within households. Small outbreaks are excluded from display. Box plots present lower and upper hinges that correspond to the first and third quartiles. The whiskers extend from the hinges to the smallest/largest values no further than 1.5*IQR. Outlying points are plotted individually. The notches extend 1.58*IQR/$\sqrt{n}$.

Scenario 2

In this scenario, we include a scaling factor to account for the difference in density between empirical-based and random mixing:

$$\begin{aligned} p_{i,1}(t) &= 1-(1-\beta_h\cdot\delta_h)^{\sum_{j\neq i\in h_i} y_{ij}I_j(t)}\cdot(1-\beta_{c,11})^{\sum_{j\notin h_i} I_{j,1}(t)}\cdot(1-\beta_{c,12})^{\sum_{j\notin h_i} I_{j,2}(t)}\\ p_{i,2}(t) &= 1-(1-\beta_h\cdot\delta_h)^{\sum_{j\neq i\in h_i} y_{ij}I_j(t)}\cdot(1-\beta_{c,21})^{\sum_{j\notin h_i} I_{j,1}(t)}\cdot(1-\beta_{c,22})^{\sum_{j\notin h_i} I_{j,2}(t)}, \end{aligned}$$

Hence, δ_h is chosen 1 for empirical-based mixing, while for random mixing it equals the network density of the simulated contact network in the realistic mixing scenario. Results from 1000 stochastic epidemic simulations are shown below.

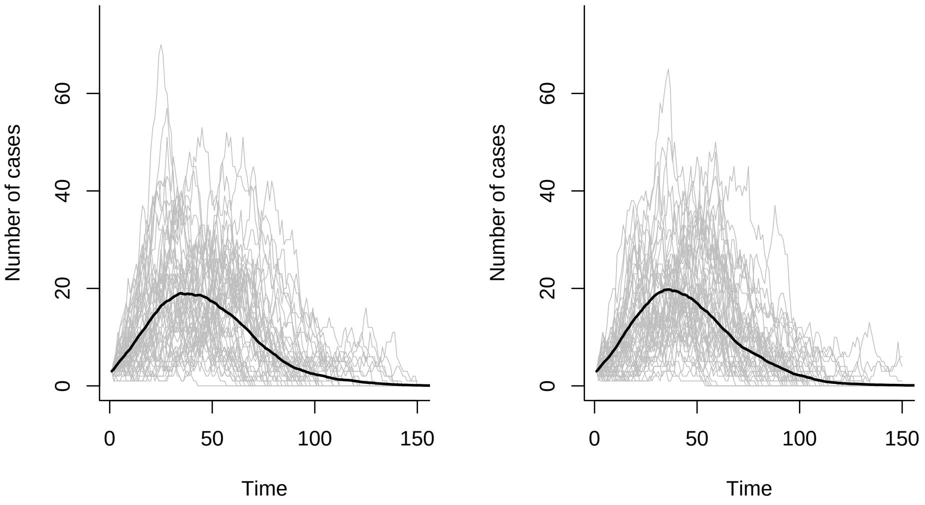

Figure S11: Total number of cases over time assuming random (left) and empirical-based mixing (right) within households including a density scaling factor. Black solid curves represent the mean and light curves correspond to 50 randomly chosen epidemics. Small outbreaks are excluded from display.

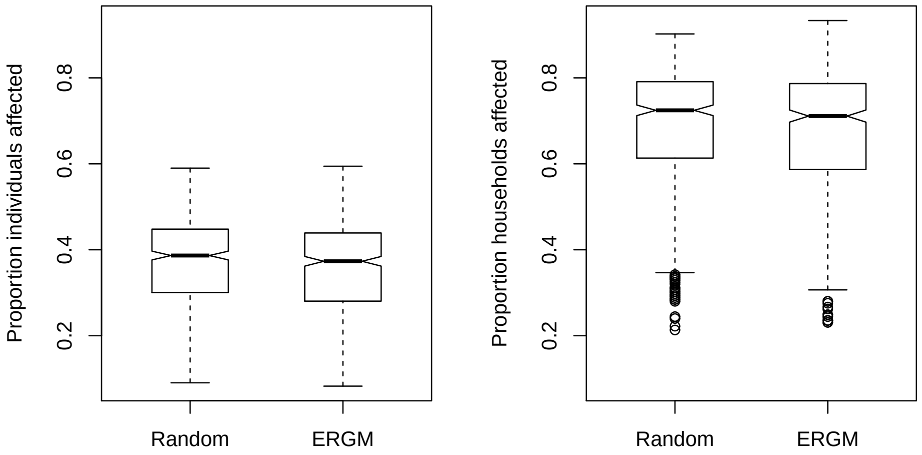

Figure S12: Final fraction of affected individuals (left) and households (right) for 1000 simulations of a stochastic SIR epidemic process on a 2-level households model assuming random and empirical-based mixing within households including a density scaling factor. Small outbreaks are excluded from display. Box plots present lower and upper hinges that correspond to the first and third quartiles. The whiskers extend from the hinges to the smallest/largest values no further than 1.5*IQR. Outlying points are plotted individually. The notches extend 1.58*IQR/$\sqrt{n}$.

Other settings

Other settings with different transmission rates, age-dependent household transmission rates, a larger community of households, or a partly vaccinated population yielded similar results (not shown).
When focussing on physical contacts with a duration of more than 4 hours (which result in a lower density network) and assuming a higher within-household transmission rate ($\beta_h = 0.3$), the resulting incidence for empirical-based mixing is lower than for random mixing regardless of correcting for the within-household density. This is shown in Figures S13-S16. The mean proportion of individuals ultimately infected and the mean proportion of households infected is larger under random mixing compared to realistic mixing: 0.78 [0.69, 0.86] vs. 0.76 [0.66, 0.83] (Wilcoxon rank sum test, p-value <0.01), and 0.91 [0.84, 0.96] vs. 0.90 [0.83, 0.95] (p-value <0.01), respectively.

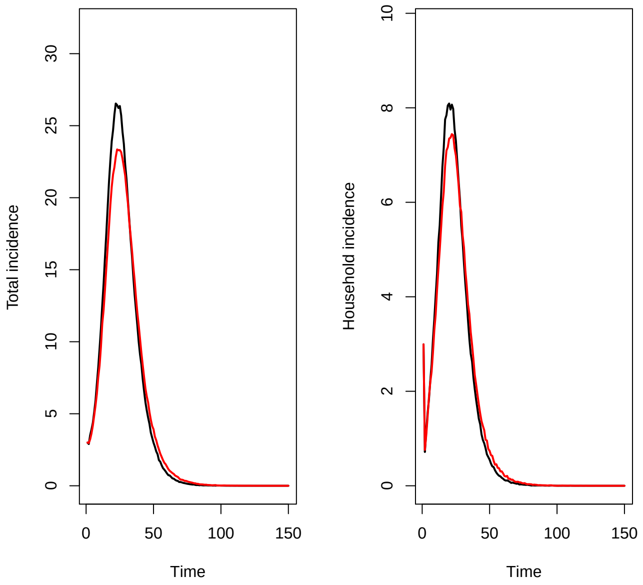

Figure S13: Mean infection incidence over time at the individual (left; number of newly infected individuals over time) and household level (right; number of newly infected households over time) assuming random (black) and empirical-based mixing (red) within households including a density scaling factor. Small outbreaks are excluded from display.

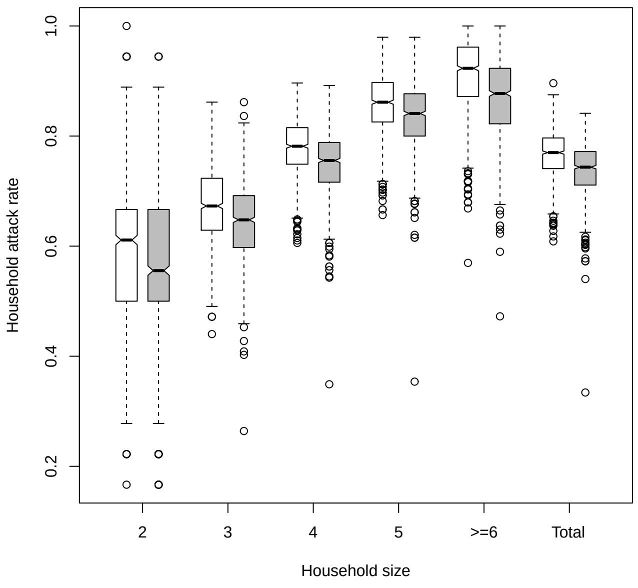

Figure S14: Household attack rates (mean proportion of infected individuals per household) by household size assuming random (white) and empirical-based mixing (gray) within households including a density scaling factor. Small outbreaks are excluded from display. Box plots present lower and upper hinges that correspond to the first and third quartiles. The whiskers extend from the hinges to the smallest/largest values no further than 1.5*IQR. Outlying points are plotted individually. The notches extend 1.58*IQR/$\sqrt{n}$.

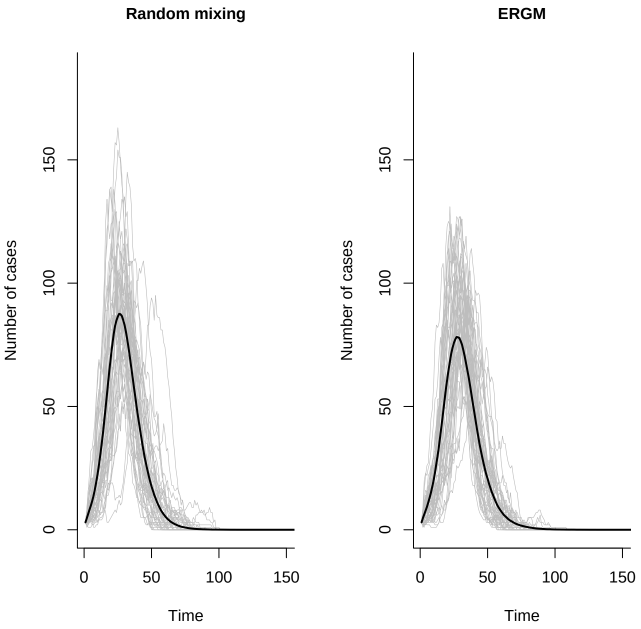

Figure S15: Total number of cases over time assuming random (left) and empirical-based mixing (right) within households including a density scaling factor. Black solid curves represent the mean and light curves correspond to 50 randomly chosen epidemics. Small outbreaks are excluded from display.

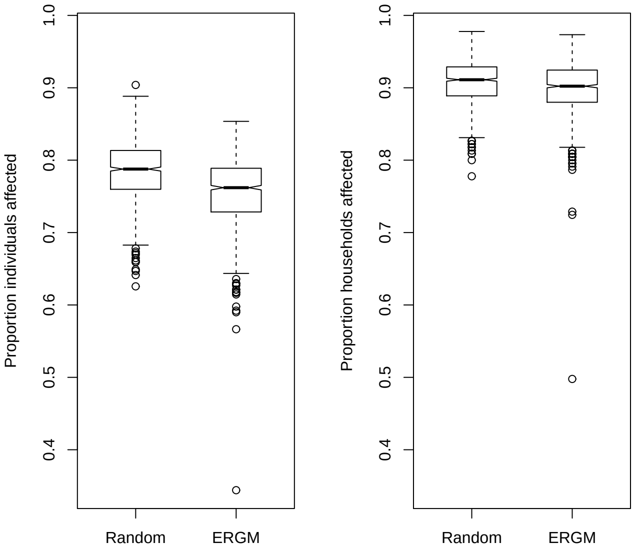

Figure S16: Final fraction of affected individuals (left) and households (right) for 1000 simulations of a stochastic SIR epidemic process on a 2-level households model assuming random and empirical-based mixing within households including a density scaling factor. Small outbreaks are excluded from display. Box plots present lower and upper hinges that correspond to the first and third quartiles. The whiskers extend from the hinges to the smallest/largest values no further than 1.5*IQR. Outlying points are plotted individually. The notches extend 1.58*IQR/$\sqrt{n}$.

Table S7: Literature-based estimates of household and community transmission parameters obtained from household final size or symptom onset data: $q_{\mathrm{HH}} = P$(escape infection from infected HH member per day) assuming an infectious period of 4 days, the household secondary attack rate (SAR) i.e. the probability of being infected by another household member during the course of the latter's infectious period, and $q_{\mathrm{com}} = P$(escape infection from community during epidemic period)= 1− CPI, where CPI is the community probability of infection. † Household size defined as the number of susceptibles in a household prior to the epidemic. * Same age definitions for children and adults as in Longini Jr. et al. (1988), distinguishing between susceptibles and infected.

Source	Data	Number of households	Household size (range)	Stratification	Household		Community	
					q_{HH}	SAR	q_{com}	CPI
Longini Jr. and Koopman (1982)	Asian influenza, Japan	$n = 42$	Size 3		0.96	0.17	0.86	0.14
	Influenza	$n = 42$	Size $4-5$		0.90	0.36	0.66	0.34
Longini Jr. et al. (1982)	1977-78 influenza A(H3N2), Tecumseh	$n = 195$	Size† $1-5$		0.96	0.15	0.87	0.13
	1975-76 influenza B, Seattle	$n = 87$	Size† $1-5$		0.97	0.13	0.83	0.17
	1977-78 influenza A(H3N2), Seattle	$n = 159$	Size NA		0.94	0.21	0.74	0.26
	1978-79 influenza A(H1N1), Seattle	$n = 93$	Size† $1-3$		0.91	0.31	0.54	0.46
Longini Jr. et al. (1988)	1977-78, 1980-81 influenza A(H3N2), Tecumseh	$n = 567$	Size† $1-5$	Child $<$ 18y	0.94	0.22	0.82	0.18
				Adult $\geq$ 18y	0.97	0.11	0.89	0.11
Addy et al. (1991)*	1977-78, 1980-81 influenza A(H3N2), Tecumseh	$n = 567$	Size† $1-5$	Child - child	0.92	0.28	0.82	0.18
				Child - adult	0.96	0.13		
				Adult - child	0.97	0.10	0.89	0.11
				Adult - adult	0.96	0.15		
Rampey et al. (1992)*	1983 rhinovirus, Tecumseh	$n = 91$	Size $3-9$	Child - child	0.95	0.17	0.63	0.37
				Child - adult	0.97	0.13		
				Adult - child	0.97	0.11	0.76	0.24
				Adult - adult	0.97	0.11		
Cauchemez et al. (2004)	1999-2000 influenza A(H3N2), France	$n = 334$	Size $2-8$	Size 2	0.87	0.43	0.92	0.08
				Size 3	0.91	0.31		
				Size 4	0.93	0.25		
				Size 5	0.94	0.21		

References

Addy, C. L., I. M. Longini Jr., and M. Haber (1991). A generalized stochastic model for the analysis of infectious disease final size data. *Biometrics 47*, 961–974.

Cauchemez, S., F. Carrat, C. Viboud, A. J. Valleron, and P. Y. Boelle (2004). A Bayesian MCMC approach to study transmission of influenza: application to household longitudinal data. *Statistics in Medicine 23*, 3469–3487.

Geyer, C. J. and E. A. Thompson (1992). Constrained Monte Carlo maximum likelihood calculations. *Journal of the Royal Statistical Society B 54*, 657–699.

Goeyvaerts, N., N. Hens, B. Ogunjimi, M. Aerts, Z. Shkedy, P. Van Damme, and P. Beutels (2010). Estimating infectious disease parameters from data on social contacts and serological status. *Applied Statistics 59*, 255–277.

Handcock, M. S. (2003). Assessing degeneracy in statistical models of social networks. Technical Report Working Paper no. 39, University of Washington, Seattle.

Hunter, D. R. (2007). Curved exponential family models for social networks. *Social Networks 29*, 216–230.

Hunter, D. R., M. S. Handcock, C. T. Butts, S. M. Goodreau, and M. Morris (2008). ergm: A package to fit, simulate and diagnose exponential-family models for networks. *Journal of Statistical Software 24*, 1–29.

Kifle, Y. W., N. Goeyvaerts, K. Van Kerckhove, L. Willem, A. Kucharski, C. Faes, H. Leirs, N. Hens, and P. Beutels (2015). Animal Ownership and Touching Enrich the Context of Social Contacts Relevant to the Spread of Human Infectious Diseases. *PloS one 10*(7), e0133461.

Kolaczyk, E. D. (2009). *Statistical Analysis of Network Data: Methods and Models*. Springer, New York.

Longini Jr., I. M. and J. S. Koopman (1982). Household and community transmission parameters from final distributions of infections in households. *Biometrics 38*, 115–126.

Longini Jr., I. M., J. S. Koopman, M. Haber, and G. A. Cotsonis (1988). Statistical inference for infectious diseases. risk-specific household and community transmission parameters. *American Journal of Epidemiology 128*, 845–859.

Longini Jr., I. M., J. S. Koopman, A. S. Monto, and J. P. Fox (1982). Estimating household and community transmission parameters for influenza. *American Journal of Epidemiology 115*, 736–751.

Morris, M., M. S. Handcock, and D. R. Hunter (2008). Specification of exponential-family random graph models: Terms and computational aspects. *Journal of Statistical Software 24*, 1–24.

Mossong, J., N. Hens, M. Jit, P. Beutels, K. Auranen, et al. (2008). Social contacts and mixing patterns relevant to the spread of infectious diseases. *PLoS Medicine 5(3)*, 381–391.

Potter, G. E. and M. S. Handcock (2010). A description of within-family resource exchange networks in a Malawian village. *Demographic Research 23*, 117–152.

Rampey, A. H., I. M. Longini Jr., M. Haber, and M. S. Monto (1992). A discrete-time model for the statistical analysis of infectious disease incidence data. *Biometrics 48*, 117–128.

Robins, G., P. Pattison, Y. Kalish, and D. Lusher (2007). An introduction to exponential random graph (p*) models for social networks. *Social Networks 29*, 173–191.

Strauss, D. and M. Ikeda (1990). Pseudolikelihood estimation for social networks. *Journal of the American Statistical Association 85*, 204–212.

Willem, L., K. Van Kerckhove, D. L. Chao, N. Hens, and P. Beutels (2012). A nice day for an infection? Weather conditions and social contact patterns relevant to influenza transmission. *PLoS ONE 7*(11), e48695.
